# Supplementary figures and images for: GLP-1RA improves diabetic renal injury by alleviating glomerular endothelial cells pyrotosis via RXRα/circ8411/miR-23a-5p/ABCA1 pathway
Source: PLoS One. 2024 Dec 2;19(12):e0314628. doi: 10.1371/journal.pone.0314628 (PMC11611192; doi:10.1371/journal.pone.0314628)

# Supplementary Figure

**Supplemental Figure 1.** ABCA1 expression in the INS group.


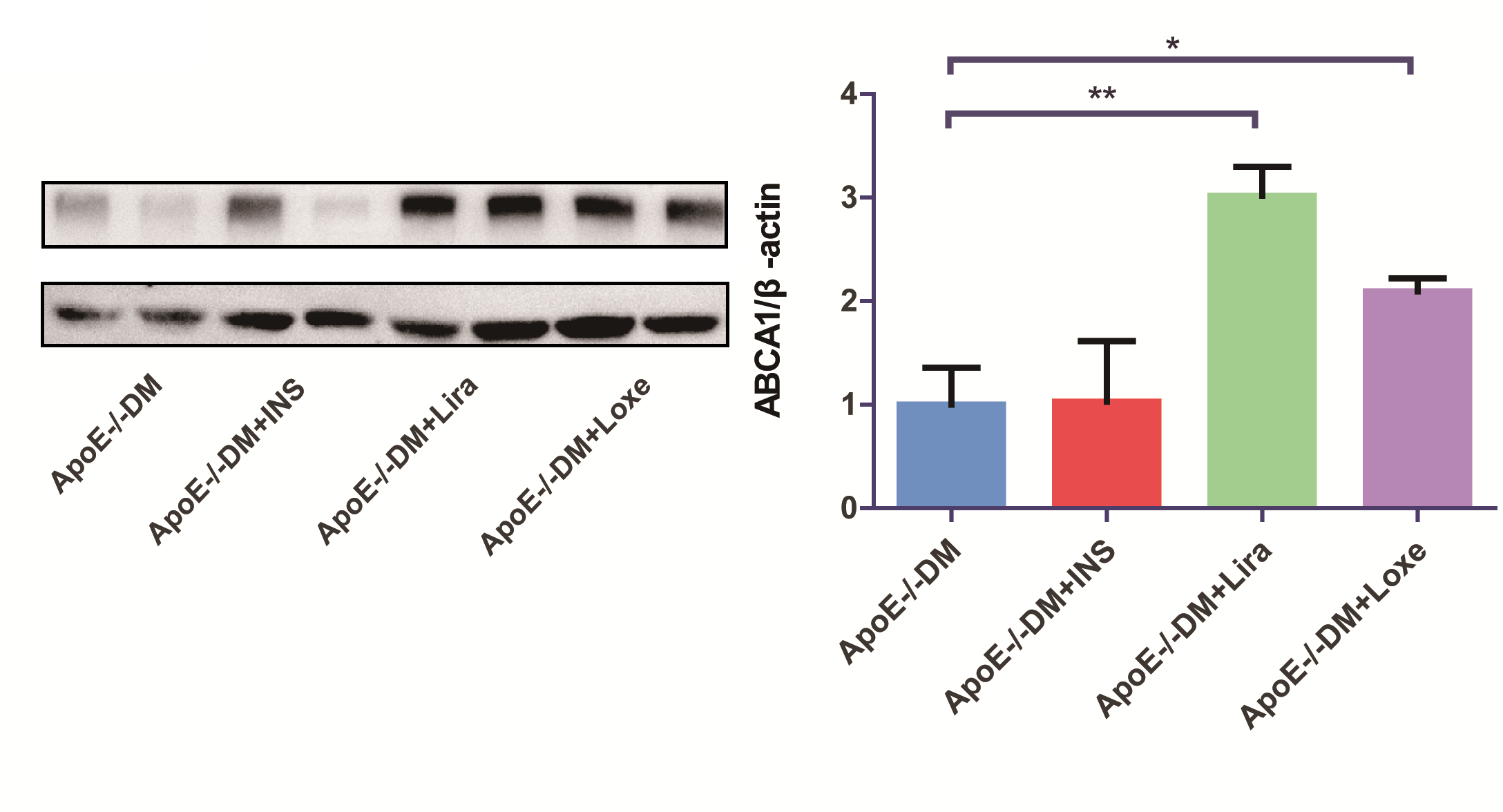

Supplement: S1 Fig — (DOCX) [file pone.0314628.s001.docx]
