# Supplementary material for: GLP-1RA improves diabetic renal injury by alleviating glomerular endothelial cells pyrotosis via RXRα/circ8411/miR-23a-5p/ABCA1 pathway
Source: PLoS One. 2024 Dec 2;19(12):e0314628. doi: 10.1371/journal.pone.0314628 (PMC11611192; doi:10.1371/journal.pone.0314628)
Supplement: S1 Raw images — (PDF) [file pone.0314628.s003.pdf]

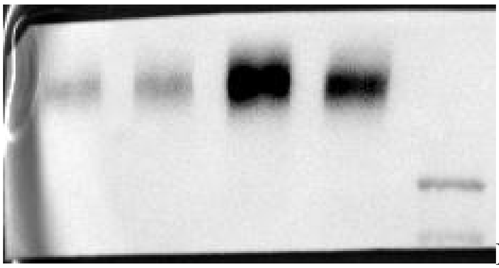LG-HG-HC-HG+HC ABCA1(254kDa)

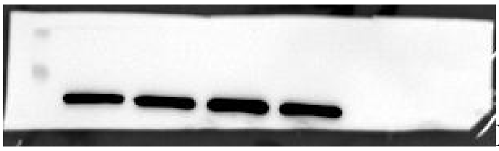LG-HG-HC-HG+HC gapdh(36kDa)

)

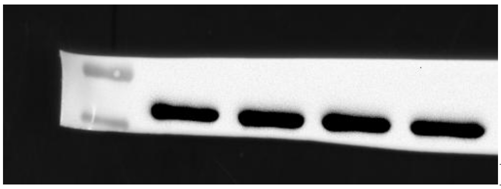LG-HG-HC-HG+HC GAPDH(36kDa)

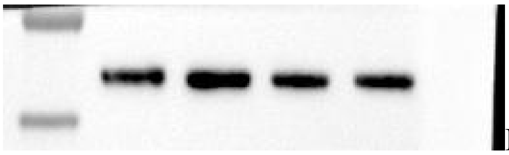LG-HG-HC-HG+HC gapdh-ABCA1(254kDa)

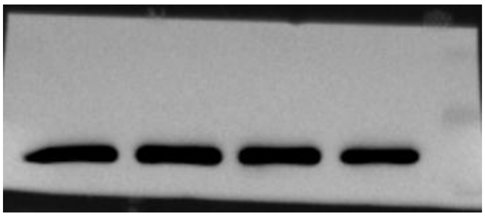LG-HG-HC-HG+HC gapdh(36kDa)

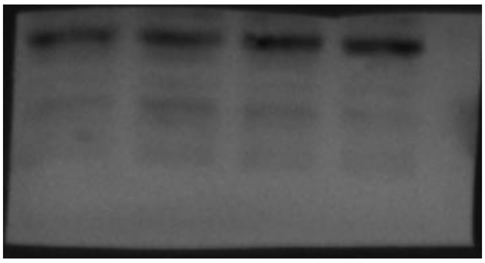LG-HG-HC-HG+HC caspase-1(20kDa)

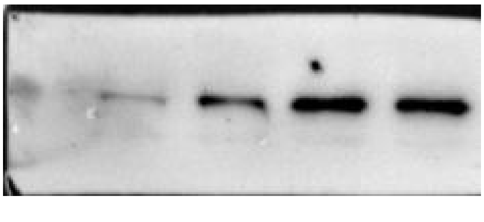LG-HG-HC-HG+HC GSDMD(53kDa)

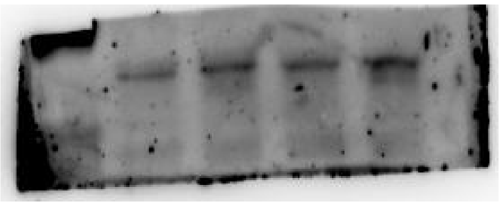 LG-HG-HC-HG+HC IL-1β(17kDa)

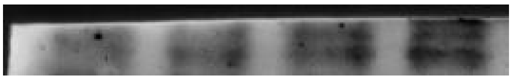 LG-HG-HC-HG+HC N-GSDMD(31kDa)

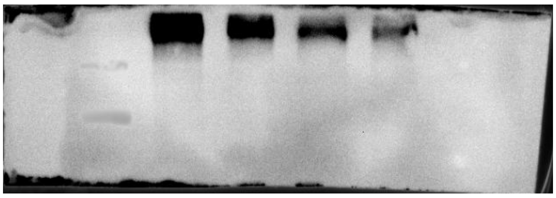 DIDS(concentration gradient)-ABCA1(254kDa)

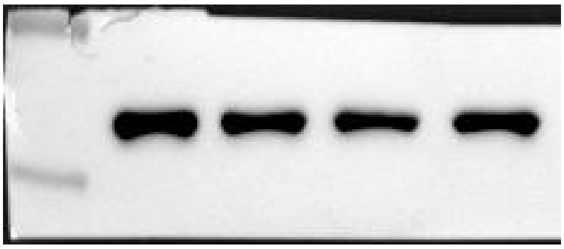 DIDS(concentration gradient)-ACTIN(43kDa)

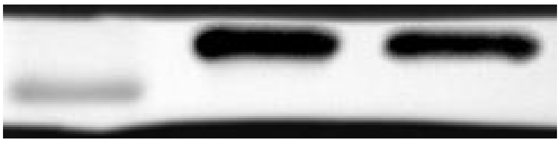 HC+HG；HC+HG+DIDS（ACTIN）(43kDa)

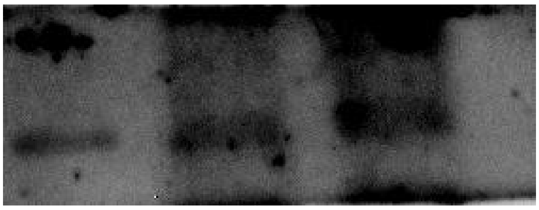 HC+HG；HC+HG+DIDS（Caspase-1）(20kDa)

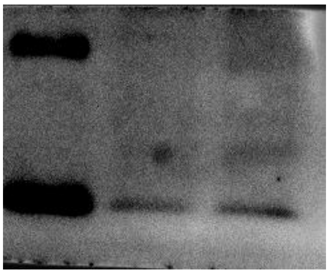 HC+HG；HC+HG+DIDS（Caspase-1）(20kDa)

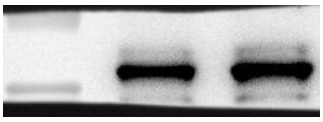
HC+HG；HC+HG+DIDS（GSDMD）(53kDa)

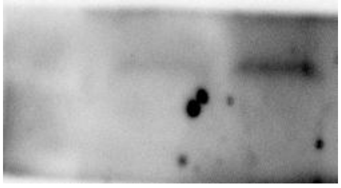
HC+HG；HC+HG+DIDS（N-GSDMD）(31kDa)

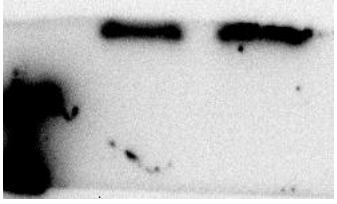
HC+HG；HC+HG+DIDS（IL-1β）(17kDa)

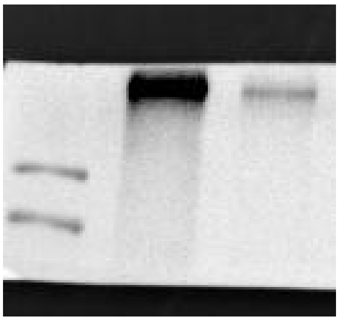
SI-circ8411 ABCA1(254kDa)

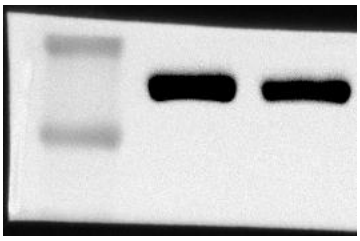
si-circ8411(HG+HC) actin(43kDa)

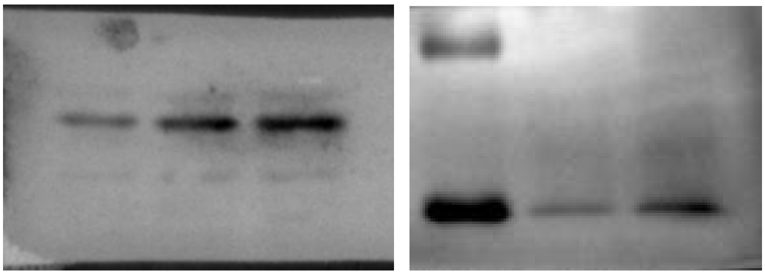
si-circ8411(HG+HC) -Caspase-1(20kDa)

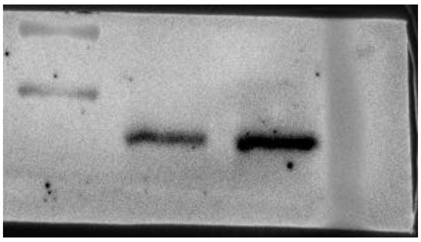
si-circ8411(HG+HC) GSDMD(53kDa)

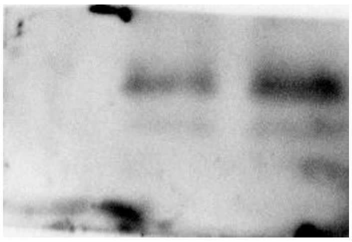
si-circ8411(HG+HC) IL-1β(17kDa)

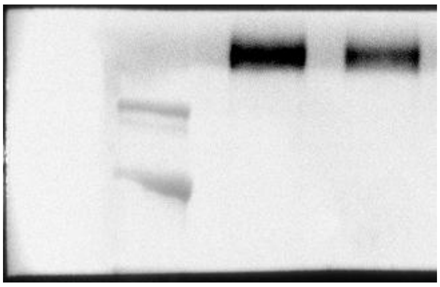
Si-circ8411(HG+HC)-ABCA1

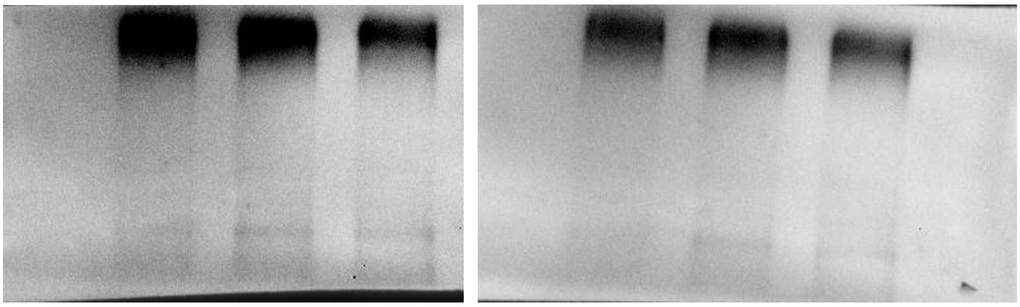

UVI3003 concentration gradient-ABCA1(254kDa)

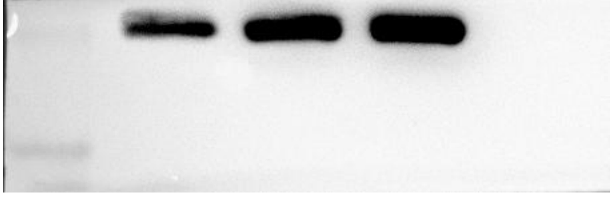
UVI3003(concentration gradient)-0-1-10 ACTIN(43kDa)

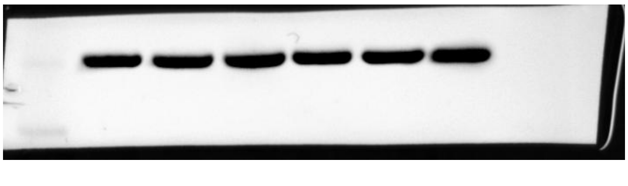
LIRA(concentration gradient）-ACTIN(43kDa)

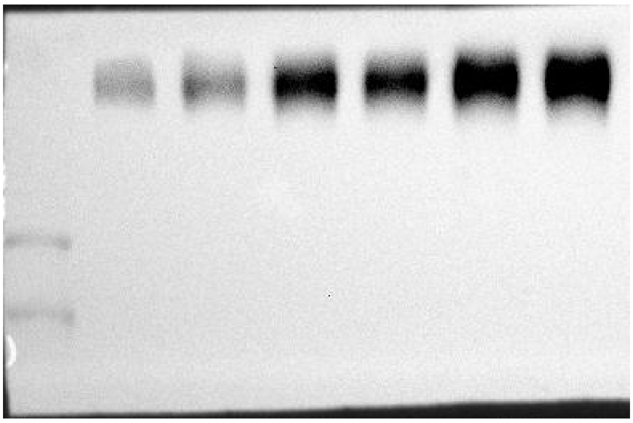 LIRA(concentration gradient)-ABCA1(254kDa)

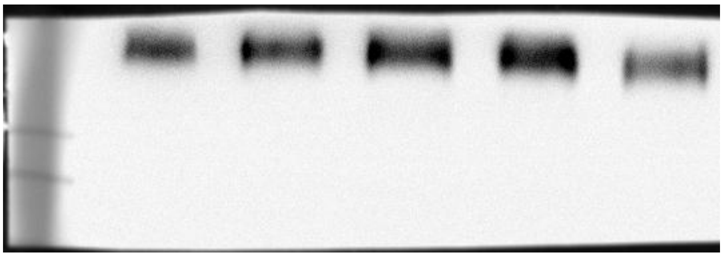 Loxe(concentration gradient)-ABCA1(254kDa)

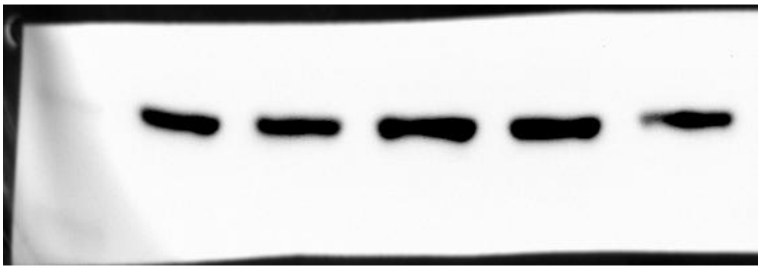 Loxe(concentration gradient)-actin

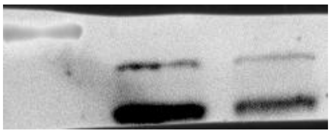 Loxe-GSDMD(53kDa)

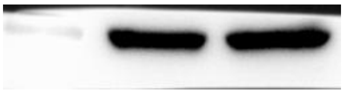 Loxe-actin(43kDa)

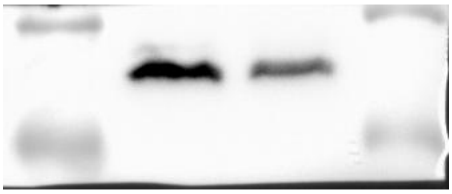 Loxe caspase-1(20kDa)

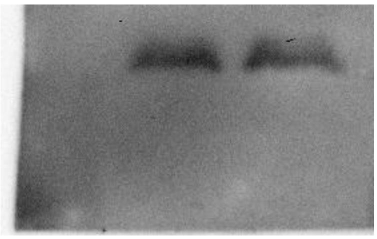

Loxe-IL-1β(17kDa)

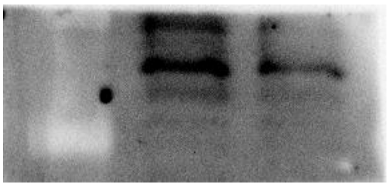

Loxe-N-GSDMD(31kDa)

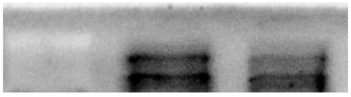

Loxe-RXRA

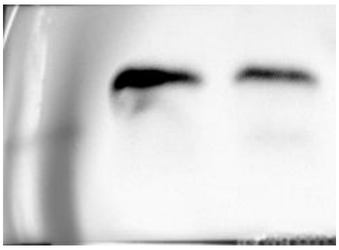

Lira-IL-1β(17kDa)

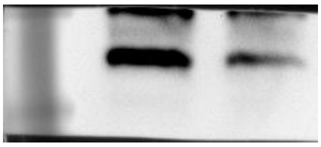

Lira-N-GSDMD(31kDa)

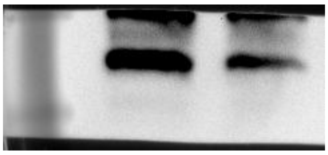

Lira-N-GSDMD(31kDa)

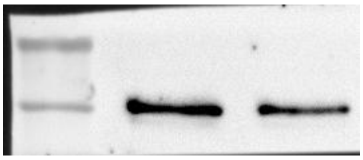

Lira-GSDMD(53kDa)

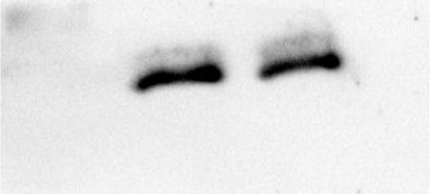

Lira-caspase-1(20kDa)

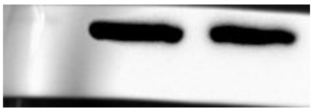
lira--actin-(43kDa)

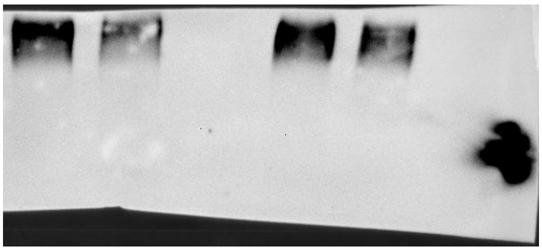
SI-CIRC8411(Lira-Loxe) ABCA1(254kDa)

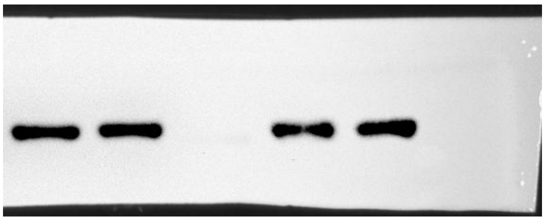
SI-CIRC8411 (Lira-Loxe) ACTIN(43kDa)

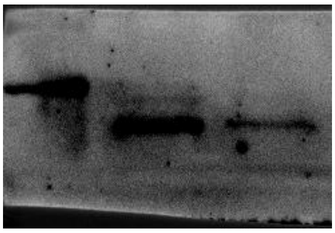
over-circ8411 (HG+HC) GSDMD(53kDa) (2)

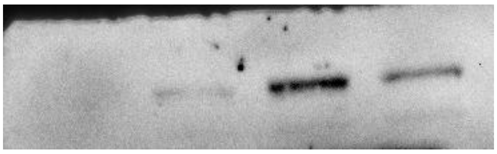
over-circ8411 (HG+HC) GSDMD(53kDa) (2)

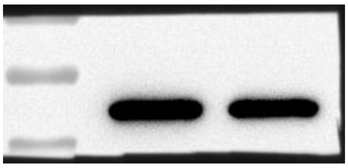
over-circ8411 (HG+HC) actin(43kDa)

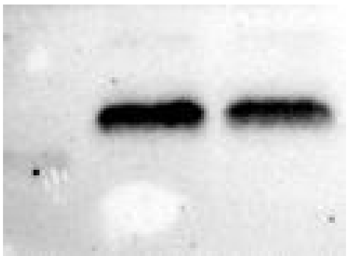
over-circ8411 (HG+HC) caspase-1(20kDa)

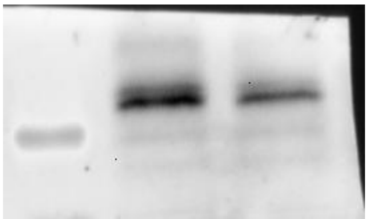

over-circ8411 (HG+HC) IL-1β(17kDa)

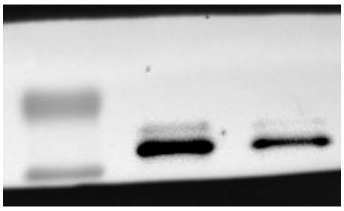

CIRC8411 OVER GSDMD(53kDa)

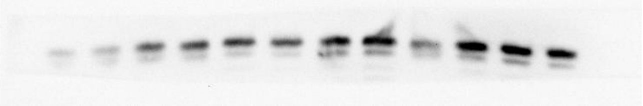

Mouse-caspase-1(20kDa)

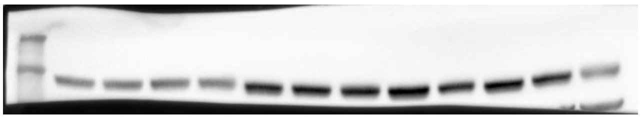

Mouse-GSDMD(53kDa)

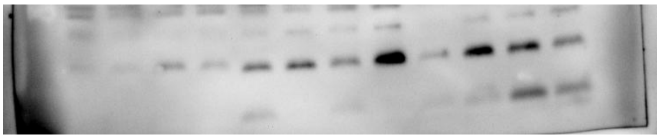

Mouse-IL-1β(17kDa)

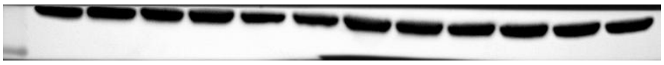

Mouse-actin-index of Pyroptosis

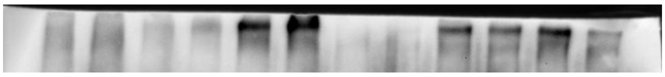

Mouse-ABCA1(254kDa)

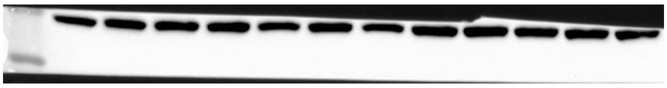

Mouse-ACTIN(53kDa)

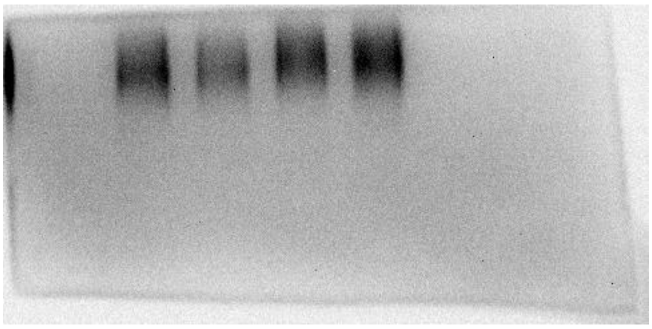

rescue-ABCA1(254kDa)

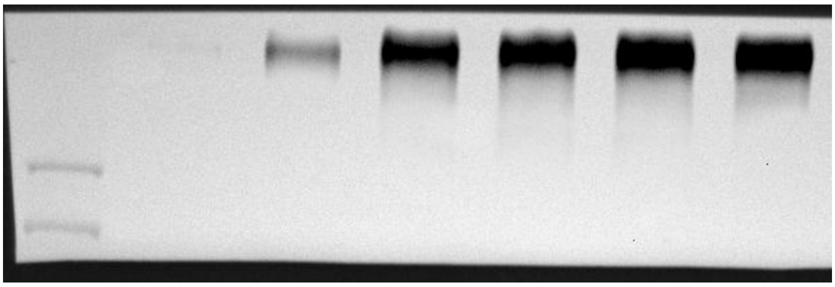

Lira-ABCA1(254kDa)

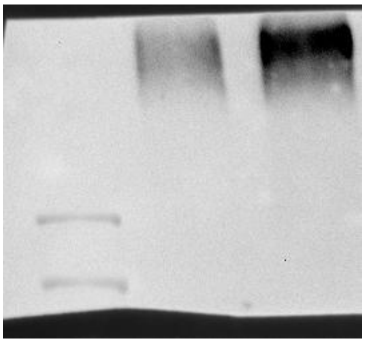

Lira-ABCA1(254kDa) (2)

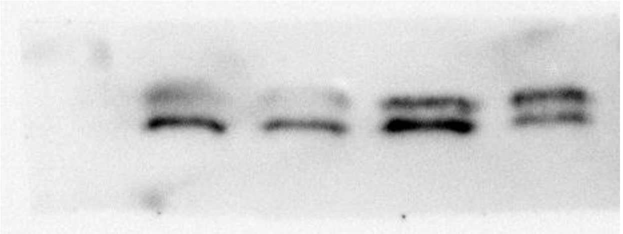

LG-HG-HC-HG+HC RXRA

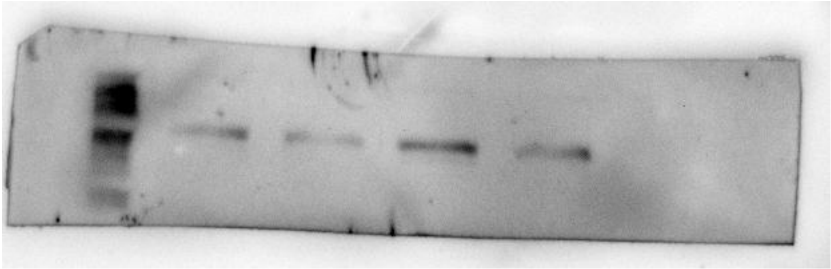

LG-HG-HC-HG+HC RXRA(2)

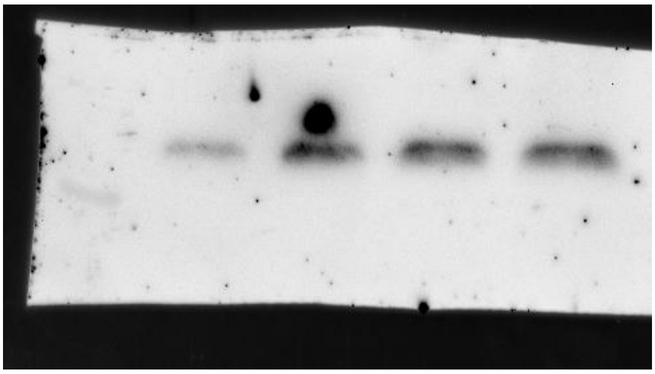

LG-HG-HC-HG+HC IL-1β

(17kDa) (2)

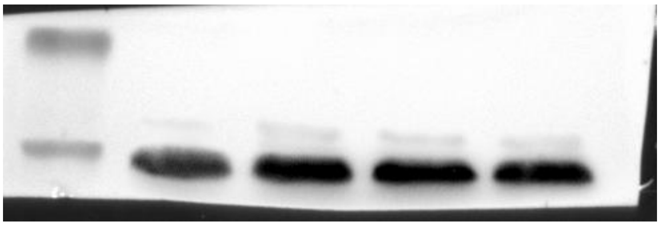 LG-HG-HC-HG+HC

GAPDH-RXRA

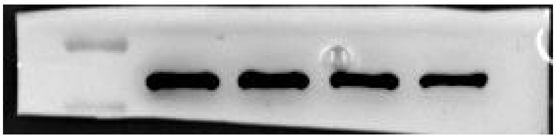 index of Pyroptosis ACITN

（LG-HG-HC-HG+HC）(43kDa)

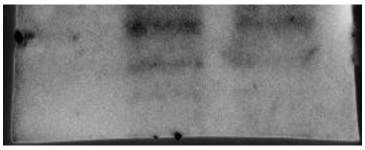 N-GSDMD(31kDa) OVER

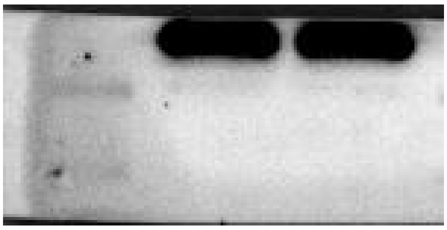 GAPDH(36kDa) OVER

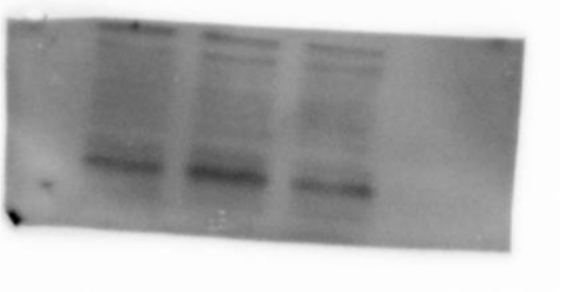 CIRC8411 OVER-caspase-1(20kDa)

(NC-VECTOR-OVER)

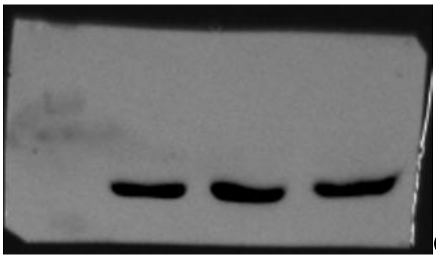CIRC8411 OVER-ACTIN

(NC-VECTOR-OVER)(43kDa)

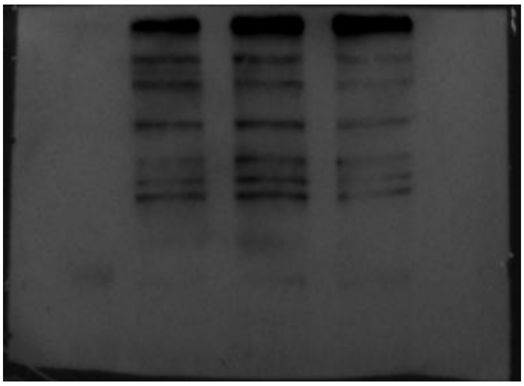 CIRC8411 OVER-ABCA1

(NC-VECTOR-OVER)
