# Supplementary material for: GLP-1RA improves diabetic renal injury by alleviating glomerular endothelial cells pyrotosis via RXRα/circ8411/miR-23a-5p/ABCA1 pathway
Source: PLoS One. 2024 Dec 2;19(12):e0314628. doi: 10.1371/journal.pone.0314628 (PMC11611192; doi:10.1371/journal.pone.0314628)
Supplement: Attachment — Submitted filename: Response to editor and reviewers.docx [file pone.0314628.s004.docx]

Dear editor and reviewers,

We would like to express our sincere gratitude to you for your efforts in handling our manuscript. We appreciate you give us the opportunity to revise our paper. We thank the editorial boards and the two reviewer’s points and suggestions to improve our work. Those comments are all valuable and very helpful for revising and improving our paper.

We have studied comments carefully and have made correction which we hope meet with approval. Based on your comment and request, we attached revised manuscript with the correction sections red marked. We also replied the reviewers’ questions point-by-point as following below. The corrections in the paper and the responds to the reviewer’s comments are as following.

Should you have any questions, please contact us without hesitate.

**Reviewer #1:**

These paper about “GLP-1 RA improves diabetic renal injury by alleviating Glomerular Endothelial Cells Pyrotosis via RXRα/circ8411/miR-23a-5p/ABCA1 pathway “suggesting that excessive hyperglycemia causes lipotoxic renal damage through the RXRα/circ8411/miR-23a-5p/ABCA1 43 pathway. Individuals suffering from hypercholesterolemia and diabetic kidney disease (DKD) may find relief by focusing on this pathway. The paper is well planned and written to put all the relevant data together. There are few minor corrections to be done. When all these minor comments are addressed, I think this paper can be considered for acceptance.

**Response:** We express our gratitude for your meticulous and constructive review, as well as your insightful comments. Your comments were highly insightful and has significantly enhanced the overall quality of our manuscript. According with your suggestions, we have made necessary revisions to the relevant section of the article. Subsequently, we present our point-by-point responses to each of your comments in the following pages.

**1. The font size throughout the paper is not the same.**

**2. The spacings in the paper should be modified to be the same throughout the paper.**

**3. It would be better if you can reduce the thickness of the error bars in all the figures.**

**4. In Fig 7K immunostaining panel please mention blue and red in the figure itself.**

**Response:** We appreciate the valuable advice provided and regret that the details of the article were not detailed enough. We have thoroughly reviewed and made revisions to this section. We've resized the body font to a uniform size, and doubled the line spacing as follows the manuscript body formatting guidelines.

**Revised version in line 419-433 (Results):**

**Figure 7. GLP-1RA alleviated kidney damage, cholesterol accumulation and pyroptosis in renal of ApoE-/-DM mice by increasing circ8411-ABCA1.** (A) HE staining of the renal of mice (×400, bar=50μm). (B) Oil red O staining of the renal of mice (×400, bar=50μm). (C) Cholesterol quantification experiment to determine the cholesterol accumulation in renal of mice (**P < 0.01 vs. WT-NC, ***P < 0.001 vs. WT-NC, #P < 0.05 vs. ApoE-/-DM, ##P < 0.01 vs. ApoE-/-DM).(D,E) Caspase-1, GSDMD, IL-1β expression of renal of mice (*P < 0.05 vs. WT-NC, **P < 0.01 vs. WT-NC, ***P < 0.001 vs. WT-NC, #P < 0.05 vs. ApoE-/-DM, ##P < 0.01 vs. ApoE-/-DM).（F) Release of LDH of renal of mice (**P < 0.01 vs. WT-NC, ##P < 0.01 vs. ApoE-/-DM). (G) Circ8411 expression of renal of mice (*P < 0.05 vs. WT-NC, ##P < 0.01 vs. ApoE-/-). (H-J) RT-qPCR, Western Blot and immunofluorescence experiment (×200, bar=50μm) were conducted to determine ABCA1(red) expression of renal of mice. Nuclei were stained with DAPI (blue). (**P < 0.01 vs. WT-NC, ***P < 0.001 vs. WT-NC, ##P < 0.01 vs. ApoE-/-, ###P < 0.001 vs. ApoE-/-, &P < 0.05 vs. WT-NC).

**Reviewer#2**

This study by Weixi Wu et al., mechanistically demonstrated the protective effect of GLP-1 RA against lipototoxity in diabetic renal injury. While this study may be of great interest to the scientific community, there are concerns to address.

**Response:** We sincerely thank for your systematic guidance of my manuscript. Your comments were highly insightful, which greatly improved the content of this article as well as its framework and writing standards. According with your suggestions, we have amended the corresponding part of the article. In the following pages are our point-by-point responses to each of the comments.

**1. L26-31. It looks to me that the aim of this study is not well described. Are the authors investigated the mechanical protective role of GLP-1 RA or investigated the cholesterol accumulation and pyroptosis in glomerular endothelial cells (GEnCs)? If it is the latter, then it does not clearly match the title. Please clarify and make it consistent with the main result that drive the title of the manuscript.**

**Response:** We appreciate the valuable advice provided and express regret for the lack of clarity in the abstract. We have thoroughly reviewed and made revisions to this section.

**Revised version in line 26-32(Methods):**

In this study, we investigated the cholesterol accumulation and pyroptosis in glomerular endothelial cells (GEnCs) and the **reno-protective** effects of GLP-1RAs using various techniques such as RT-qPCR, oil red O staining，Western blotting, and LDH activity assay. We also employed circRNA microarrays, bioinformatics analysis, gain and loss of function experiments, rescue experiments, and luciferase assay to identify the ceRNA mechanism involved. C57BL/6J, ApoE-/- mice were used for in vivo experiments.

**2. L51-53. Please improve the logical flow of the manuscript. I suggest the authors to write the second sentence first, then the first after: "Diabetic microvascular complications including DKD, leads to end stage renal disease [1]. In fact, globally, diabetic kidney disease (DKD) is a serious health and economic problem [2].**

**Response:** Thank you for bringing this to our attention. We sincerely appreciate your careful reading. We have diligently examined and refined the manuscript, encompassing the introduction through to the references.

**Revised version in line 52-54(Introduction):**

Diabetic microvascular complications include diabetic kidney disease (DKD), which leads to end-stage renal disease [1]. Globally, DKD is a serious health and economic problem [2].

**Revised version in line 606-613(****References):**

1.	de Boer IH, Khunti K, Sadusky T et al. (2022) Diabetes management in chronic kidney disease: a consensus report by the American Diabetes Association (ADA) and Kidney Disease: Improving Global Outcomes (KDIGO). Kidney Int 102: 974-989. <https://doi.org/10.1016/j.kint.2022.08.012>

2.	Deng Y, Li N, Wu Y et al. (2021) Global, Regional, and National Burden of Diabetes-Related Chronic Kidney Disease From 1990 to 2019.Front Endocrinol (Lausanne) 12: 672350. <https://doi.org/10.3389/fendo.2021.672350>

**3.L53-55. Please rewrite the following "The current approaches to preventing or treating DKD have limited effectiveness, such as controlling blood glucose levels, blood pressure, and urinary protein excretion" as: "****The current approaches to prevent or treat DKD, which includes controlling blood glucose levels, blood pressure, and urinary protein excretion, have limited effectiveness. This limited effectiveness can be attributed to the lack of knowledge regarding the pathophysiological mechanism involved.**

**Response:** We sincerely appreciate your careful reading. We think this is an excellent suggestion. We have re-edited the paragraph according to your suggestions.

**Revised version in line 54-57(Introduction):**

The current approaches to prevent or treat DKD, which includes controlling blood glucose levels, blood pressure, and urinary protein excretion, have limited effectiveness. This limited effectiveness can be attributed to the lack of knowledge regarding the pathophysiological mechanism involved[3].

**4.L58-59. hard to understand sentence: not [... and triglyceride and cholesterol accumulated in the kidney of diabetes models is thought possibly contribute to DKD pathogenesis] BUT [...** **and accumulation of triglyceride and cholesterol in the kidney of diabetes models potentially contribute to DKD pathogenesis] or [... and accumulation of triglyceride and cholesterol in the kidney of diabetes models is thought to contribute to DKD pathogenesis].**

**Response:** Thank you for your valuable input. We have re-edited the paragraph according to your suggestions.

**Revised version in line 58-60(Introduction):**

Studies have confirmed that abnormal lipid metabolism is strongly associated with DKD, and accumulation of triglyceride and cholesterol in the kidney of diabetes models potentially contribute to DKD pathogenesis[4-6].

**5.L69-70. Hard-to-understand sentence [To our knowledge, ATP-binding cassette transporter A1(ABCA1), which serve as transporters that promote cholesterol efflux from cell]. Please, rewrite it simply but accurately, or improve punctuation for a less confusing writing.**

**Response:** Thank you for your valuable input. We apologize for the poor language of our manuscript. We have now worked on both language and readability and have also involved native English speakers for language corrections. We really hope that the flow and language level have been substantially improved. We have re-edited the paragraph according to your suggestions.

**Revised version in line 71-73(Introduction):**

The ATP-binding cassette transporter A1 (ABCA1) acts as a transporter that facilitates the removal of cholesterol from cells, by promoting cholesterol efflux.

**6. "renoprotective or "reno-protective"? Please be consistent.**

**Response:** We feel sorry for our carelessness. In our resubmitted manuscript, the typo is revised. Thanks for your correction.

**Revised version in line 23-25(Background):**

Furthermore, the mechanisms underlying any potential reno-protective effects of GLP-1 receptor agonists (GLP-1RAs) have not yet been established.

**Revised version in line 26-29(Methods):**

In this study, we investigated the cholesterol accumulation and pyroptosis in glomerular endothelial cells (GEnCs) and the reno-protective effects of GLP-1RAs using various techniques such as RT-qPCR, oil red O staining，Western blotting, and LDH activity assay.

**Revised version in line 89-92(Introduction):**

Taken together, we hypothesize that the GLP-1RA could exerts its reno-protective effects by regulating lipotoxicity-induced GEnCs pyroptosis via regulating ABCA1 in DKD and circRNAs may be involved in this procedure.

**Revised version in line 554-556(Results):**

In our study, we found that liraglutide and loxenatide has reno-protective and lipid-regulating effect in ApoE-/- DM mouse.

**Revised version in line 578-581(Discussion)**

More importantly, we first identify that liraglutide and loxenatide achieves reno-protection by reducing cholesterol accumulation and [pyroptosis](C:/Users/49172/AppData/Local/youdao/dict/Application/8.9.6.0/resultui/html/index.html#/javascript:;) at least in part through RXRα/circ8411/miR-23a-5p/ABCA1 pathway under diabetic conditions.

**7.** **L65. no need to repeat " interleukin-18" just right IL-18.**

**Response:** Thank you for your valuable input. We have re-edited the paragraph according to your suggestions.

**Revised version in line 65-68(Introduction):**

Activating caspase-1 triggers the process of pyroptosis, inherently inducing inflammation and releasing pro-inflammatory factors, such as interleukin-1β (IL-1β), IL-18, lactate dehydrogenase (LDH) and the activation of a stronger inflammatory response[10].

**8. It is suggested that the authors provide Ethics numbers or a copy of university ethical approval statement with institutional stamp.**

**Response:** Thank you for your valuable input. We will upload a a copy of university ethical approval statement with institutional stamp.

**9.** **L92. Suggested to change "Cellular experiments" with "****Cell culture experiments"**

**Response:** Thank you for your valuable input. We have re-edited the paragraph according to your suggestions.

**Revised version in line 94(Materials and methods):**

Cell culture experiments

**10. L94-97. The cell cultures were divided into different growth conditions, including low glucose (LG, 5.5 mmol/L D-glucose ), high glucose (HG, 25.5 mmol/L D-glucose), high cholesterol (HC, 400 µg/ml watersoluble cholesterol) (Sigma, United States)], and HG combined HC (25.5 mmol/L+400 µg/ml).**

**Response:** Thank you for your valuable input. Based on your comments, we have made the corrections and attached it below.

**Revised version in line 96-99(Cell culture experiments):**

The cell cultures were divided into different growth conditions, including low glucose (LG, 5.5 mmol/L D-glucose ), high glucose (HG, 25.5 mmol/L D-glucose), high cholesterol (HC, 400 µg/ml watersoluble cholesterol) (Sigma, United States)], and HG combined HC (25.5 mmol/L+400 µg/ml).

**11. L198. [Values are shown as "mean + SD"]**

**Response:** Thank you for your valuable input. We have re-edited the paragraph according to your suggestions.

**Revised version in line 210(Statistical Analysis):**

Values are shown as the "mean + SD".

**12. Captions in Figures only described what experiments have been performed, but not what each figure actually tells as information that answers the scientific question (the aim) of the study. Figures are meaningless/speechless. Figures, supported with captions should stand alone and provide enough information in order, such that we do not need to read the main text to get the main idea delivered by a figure.**

**Response:** Thank you for your valuable input. We've re-edited the captions.

**Revised version in line 258-260;272-273(Results):**

**Figure 1. High glucose exacerbates intracellular cholesterol accumulation and pyroptosis under cholesterol load in GEnCs.** (A) CCK-8 assay was used to evaluate the influence of water-soluble cholesterol on GEnCs viability (*P < 0.05 vs. Control; **P < 0.01 vs. Control,***P < 0.001 vs. Control). 25.5 mmol/L glucose and 400 mg/ml cholesterol were found to be the best intervention concentrations for HK-2 cells. (B) Oil red O staining of GEnCs treated with different concentrations of cholesterol (×400, bar=50μm). (C) Cholesterol quantification experiment was conducted to determine the content of intracellular cholesterol (*P < 0.05 vs. Control; **P < 0.01 vs. Control). (D) Expression of ABCA1 in GEnCs with different cholesterol concentrations (**P < 0.01 vs. Control,***P < 0.001 vs. Control). (E,F) Expression of ABCA1 after intervention with different glucose concentrations without cholesterol and under cholesterol load (*P < 0.05 vs. Control). (G) Oil red O staining of GEnCs treated with different concentrations of cholesterol and glucose (×400, bar=50μm). (H) Cholesterol quantification experiment was conducted to determine the content of intracellular cholesterol (*P < 0.05 vs. LG,**P < 0.01 vs. LG; ***P < 0.001vs. LG). (I) RT-qPCR analysis to determine ABCA1 mRNA expression (***P < 0.001 vs. LG; #P < 0.05 vs. HC). (J) Western Blot analysis to determine ABCA1 protein expression (***P < 0.001 vs. LG; #P < 0.05 vs. HC). ABCA1 levels decreased in the HG group and increased significantly in the HC group. (K) Immunofluorescence experiment was conducted to determine the expression of ABCA1 (×200, bar=100μm).

**Revised version in line 280-281(Results):**

**Figure 2. HG exacerbates intracellular cholesterol accumulation and pyroptosis under cholesterol load.** (A,B) RT-qPCR and Western Blot analysis to determine expressions of caspase-1, GSDMD, IL-1β in GEnCs under the condition of high glucose or/and high cholesterol (*P < 0.05 vs. LG,**P < 0.01 vs. LG; #P < 0.05 vs.HC, ##P < 0.01 vs.HC). Significant increase of pyroptosis markers were conserved in HG+HC than in HG or HC. (C) The release of LDH of GEnCs treated with different concentrations of cholesterol and glucose ((*P < 0.05 vs. LG,**P < 0.01 vs. LG; ##P < 0.01 vs.HC). (D,E)RT-qPCR and Western Blot analysis to determine ABCA1 expression of GEnCs treated with different concentrations of DIDS (*P < 0.05 vs. Control,***P < 0.001 vs. Control). (F) Oil red O staining of GEnCs treated with DIDS (×400, bar=50μm). (G) Cholesterol quantification experiment was conducted to determine the content of intracellular cholesterol (*P < 0.05 vs. Control). (H,I) RT-qPCR and Western Blot analysis to determine expressions of caspase-1, GSDMD, IL-1β in GEnCs treated with DIDS (*P < 0.05 vs. Control; **P < 0.01 vs. Control, ***P < 0.001 vs. Control). (J) Release of LDH of GEnCs treated with DIDS (****P < 0.001 vs. Control).

**Revised version in line 316-317;323-324(Results):**

**Figure 3. Circ8411 is involved in the regulation of ABCA1.** (A) The heatmap for the differential expression of circRNAs. (B) RT-qPCR analysis to determine the expression of four candidate LncRNAs (*P < 0.05 vs. HC; **P < 0.01 vs. HC). (C) Circ8411 expression of GEnCs treated with cholesterol and glucose (***P < 0.001 vs. LG, ###P < 0.001 vs. HC). The level of circ8411 increased in HC compared to LG or HG, but decreased in HG+HC. (D) RT-qPCR to determine the knockdown efficiency of circ8411 (*P < 0.05 vs. si-NC). (E,F) Oil red O staining of GEnCs (×400, bar=20μm) and cholesterol quantification experiment to determine the cholesterol accumulation in cells transfected with siRNA. (G-I) RT-qPCR, Western Blot and immunofluorescence experiment (×200, bar=50μm) were conducted to determine expression of ABCA1 in cells transfected with siRNA (*P < 0.05 vs. NC-si; **P < 0.01 vs. NC-si). Using siRNA to knock down circ8411 under LG or HG+HC conditions significantly decreased ABCA1 expression. (J.K) RT-qPCR and Western Blot analysis to determine expressions of caspase-1, GSDMD, IL-1β in GEnCs treated with siRNA (*P < 0.05 vs. NC-si; **P < 0.01 vs. NC-si, ***P < 0.001 vs. NC-si). (L) Release of LDH of GEnCs treated with siRNA (*P < 0.05 vs. NC-si). (M) RT-qPCR analysis to determine the overexpression efficiency of circ8411 (***P < 0.001 vs. vector); (N,O) Oil red O staining of GEnCs (×400, bar=20μm) and cholesterol quantification experiment to determine the cholesterol accumulation in cells transfected with circ8411-pcDNA3.1 plasmid (**P < 0.01 vs. vector). (P,Q,S) RT-qPCR, Western Blot and immunofluorescence experiment (×200, bar=50μm) were conducted to determine the expression of ABCA1 in cells transfected with the circ8411-pcDNA3.1 plasmid (*P < 0.05 vs. vector, **P < 0.01 vs. vector). (R,S) RT-qPCR and Western Blot analysis to determine expressions of caspase-1, GSDMD, IL-1β in GEnCs treated with circ8411-pcDNA3.1 plasmid (*P < 0.05 vs. LG; **P < 0.01 vs. LG). (T) Release of LDH of GEnCs treated with circ8411-pcDNA3.1 plasmid (*P < 0.05 vs. vector).

**Revised version in line 358;365-366(Results):**

**Figure 4. The circ8411 regulates ABCA1 expression by regulating miR-23a-5p.**(A) RT-qPCR analysis to determine miR-23a-5p in the GEnCs undering glucose and cholesterol. MiR-23a-5p levels decreased in HC but increased in HG+HC. (B,C) RT-qPCR analysis to determine miR-23a-5p expression with the knockdown and overexpression of circ8411. (**P < 0.05 vs. Control). (D,E) RT-qPCR analysis to determine the overexpression efficiency of miR-23a-5p and the expression of ABCA1 in cells transfected with the miR-23a-5p mimic (**P < 0.05 vs. Control). (F,G) Rescue experiments to determine the regulatory effect of circ8411/miR-23a-5p/ABCA1 in GEnCs (*P < 0.05 vs. mimic NC; #P < 0.05 vs. miR-23a-5p mimic with circ8411 vector). Over-expressing miR-23a-5p suppressed ABCA1 expression, but over-expressing circul8411 reversed this suppression. (H) Luciferase reporter assay on GEnCs co-transfected with circ8411-3’UTR-WT or circ8411-3’UTR-MUT and miR-23a-5p mimics or NC (**P < 0.01 vs. Circ8411-WT+NC group).

**Revised version in line 388-389;391-392(Results):**

**Figure 5. High glucose affects circ8411 down-regulation through RXRs.** (A) RT-qPCR analysis to determine expression of RXRα, CEBP/α, Hif-1a in the GEnCs undering glucose and cholesterol (*P < 0.05 vs. Control, **P < 0.01 vs. Control). (B) Western Blot showed the expression of RXRα GEnCs under different intervention of glucose and cholesterol (*P < 0.05 vs. Control). (C) RXRα expression of GEnCs treated with different concentrations of UVI3003 (*P < 0.05 vs. Control). Clear reduction of RXRα in cells treated with 10umol/L of UVI3003. (D) Circ8411 expression of GEnCs treated with UVI3003. (E) ABCA1 expression of GEnCs treated with UVI3003 (*P < 0.05 vs. Control). Inhibiting RXRα led to decreased circ8411α and ABCA1 levels.

**Revised version in line 415-417;421-422(Results):**

**Figure 6. GLP-1RA attenuated cholesterol accumulation and pyroptosis in GEnCs by increasing RXRα-circ8411-miR-23a-5p-ABCA1.** (A,B) CCK-8 assay was used to evaluate the influence of liraglutide and loxenatide on GEnCs viability (***P < 0.001 vs. Control). ABCA1 expression was significantly higher in GEnCs treated with 1000nmol/L liraglutide and 80nmol/L loxenatide in high glucose and high cholesterol conditions. (C,D) ABCA1 expression in the GEnCs treated with different concentrations of liraglutide and loxenatide (*P < 0.05 vs. Control; **P < 0.01 vs. Control). (E-H) Oil red O staining of GEnCs (×400, bar=50μm) and cholesterol quantification experiment to determine the cholesterol accumulation in cells treated with liraglutide and loxenatide (*P < 0.05 vs. Control). Liraglutide and loxenatide treatment decreases cholesterol accumulation. (I,J,L,M) Expressions of caspase-1, GSDMD, IL-1β in GEnCs treated with liraglutide and loxenatide (*P < 0.05 vs. Control; **P < 0.01 vs. Control). (K,N) Release of LDH of GEnCs treated with liraglutide and loxenatide (**P < 0.01 vs. Control). (O-T) Expressions of RXRα, circ8411 and miR-23a-5p of GEnCs treated with liraglutide and loxenatide (*P < 0.05 vs. Control, **P < 0.01 vs. Control).(U-V) Western Blot was performed to determine the ABCA1 expression in GEnCs treated with siRNAof circ8411 and GLP-1RA(*P < 0.05 vs. Control).

**13.** **205-207. Hard to understand sentence, hampering the result presentation.**

**Response:** Thank you for your valuable input. We have re-edited the paragraph according to your suggestions.

**Revised version in line 218-222(Results):**

CCK8 assay was used to establish the optimal concentration and incubation time of the cholesterol and glucose. Based on the results of the CCK8 assay, 25.5 mmol/L glucose concentrations and 400 mg/ml cholesterol were determined to be optimal intervention concentrations for HK-2 cells，and the optimal intervention time was 24 hours.

**14.** **207-209. The authors said that [The Oil red O staining and total cholesterol quantification showed that cholesterol accumulation "was not remarkable" in HG compared with LG, but increased under HC], but latter said that [these results showed that high glucose might exacerbate the accumulation of intracellular cholesterol under the load of cholesterol]. It looks contradictory to me. Rather, the results shows that the combination of HG and HC is required/associated with cholesterol accumulation, but not only HG alone. However, when we read the following data, it showed that HG alone or together with HC induce cholesterol accumulation and proinflammatory response, through inhibition of ABCA1. The authors should take care of the following sentence [The Oil red O staining and total cholesterol quantification showed that cholesterol accumulation "was not remarkable" in HG compared with LG, but increased under HC], which, I fell, is contradictory.**

**Response:** We express our sincere gratitude for your meticulous and insightful comments. I apologize for not clarifying this issue in the manuscript. This paragraph mainly aims to explain that high concentrations of glucose alone can worsen lipid deposition inside cells, but the impact is not as significant as when high concentrations of glucose are combined with high concentrations of cholesterol during cellular intervention. Furthermore, it aims to indicate that high glucose can exacerbate lipid deposition inside cells under a cholesterol burden. Based on your comments, we have made the corrections and attached it below.

**Revised version in line 221-224(Results):**

Oil Red O staining and total cholesterol quantification indicate that, compared to the LG group, cholesterol accumulation increases in the HG group, but the extent of increase is not as significant as that observed in the HC group (Figure 1A-F).

**15.L232. rewrite as follows "****This heat map presented in Figure 3A displays the microarray data of aberrantly expressed circRNAs."**

**Response:** Thank you for your valuable input. We have re-edited the paragraph according to your suggestions.

**Revised version in line 281-282(Results):** This heat map presented in Figure 3A displays the microarray data of aberrantly expressed circRNAs.

**16.** **I will stop the review at this stage, as and suggest the authors, to take the following together with what precede into accounts:**

**Overall: I would suggest the authors to improve the writeup by fixing the grammatical errors and syntax, and doublechecking the punctuations. This manuscript suffers from serious logical flow, from a sentence to another, and from two consecutive paragraph; this needs to be taken care of. I suggest the authors to seek for help from a scientific, with professional and proficient English.**

**The methods section needs to be written with clear details that can allow repeatability of the study. English syntax hampers understanding of the shared science. The authors should redo the data analysis. If contradictory results are found, need to stand as limitations of the study or provide better scientific explanations**

**Response:** We apologize for the presence of grammatical and typographical errors in our article, as they should have been avoided. We express our gratitude for the corrections provided by the expert. In order to refine the language and rectify any remaining errors, we have submitted them to a professional editing service. We attached revised manuscript with the correction sections red marked. According with your suggestions, we have provided a more detailed description of methods section in the article. We have re-analyzed all the results and the results are consistent with the previous analysis.

Once again, thank you very much for your constructive comments and suggestions which would help us both in English and in depth to improve the quality of the paper.

Yours sincerely,

Pei Yu

2024/6/30
