# Supplementary material for: GLP-1RA improves diabetic renal injury by alleviating glomerular endothelial cells pyrotosis via RXRα/circ8411/miR-23a-5p/ABCA1 pathway
Source: PLoS One. 2024 Dec 2;19(12):e0314628. doi: 10.1371/journal.pone.0314628 (PMC11611192; doi:10.1371/journal.pone.0314628)
Supplement: Attachment — Submitted filename: Response to editor and reviewers.docx [file pone.0314628.s005.docx]

Dear editor and reviewers,

We would like to express our sincere gratitude to you for your efforts in handling our manuscript. We appreciate you give us the opportunity to revise our paper. We thank the editorial boards and the reviewer’s points and suggestions to improve our work. Those comments are all valuable and very helpful for revising and improving our paper.

We have studied comments carefully and have made correction which we hope meet with approval. Based on your comment and request, we attached revised manuscript with the correction sections red marked. The corrections in the paper and the responds to the reviewer’s comments are as following.

Should you have any questions, please contact us without hesitate.

**Reviewer #3: I appreciate your efforts in revising the manuscript, but several areas still require significant attention. Language and grammar issues persist throughout, and I strongly recommend professional editing to enhance clarity. Additionally, your responses to the reviewers' comments lack specificity. Consistent terminology (e.g reno-protective) and figure caption (too descriptive) are also necessary. A clearer point-by-point response will be beneficial in guiding your revisions (and do not need to cite the tools)**

**Response:** We apologize for the presence of grammatical and typographical errors in our article, as they should have been avoided. We express our gratitude for the corrections provided by the expert. In order to refine the language and rectify any remaining errors, we have submitted them to a professional editing service. We attached revised manuscript with the correction sections red marked. Based on your suggestions, we have simplified the figure caption.

**Revised version in line 26,29(Abstract) :**

Lipotoxicity has been implicated in diabetic kidney disease (DKD). However, the role of high glucose levels in this process and the underlying mechanisms of renal protective effects of GLP-1 receptor agonists (GLP-1RAs) remain unclear.

To investigate cholesterol accumulation, pyroptosis in glomerular endothelial cells (GEnCs), and the renal protective effects of GLP-1RAs, we employed various techniques, including RT-qPCR, Oil Red O staining, Western blotting, lactate dehydrogenase (LDH) activity assays, circRNA microarrays, bioinformatics analysis, gain- and loss-of-function experiments, rescue experiments, and luciferase assays. Additionally, in vivo experiments were conducted using C57BL/6J and ApoE-/- mice.

**Revised version in line 613(Discussion) :**

In our study, we observed that liraglutide and loxenatide exhibited renal protective and lipid-regulating effects in ApoE-/-DM mice. These GLP-1RAs decreased cholesterol accumulation in ApoE-/-DM mice and GEnCs treated with high glucose and high cholesterol.

**Revised version in Results :**

**Figure 1. High glucose exacerbates intracellular cholesterol accumulation and pyroptosis under cholesterol load in GEnCs.** (A) CCK-8 assay was used to evaluate the influence of water-soluble cholesterol on GEnCs viability (*P < 0.05 vs. Control; **P < 0.01 vs. Control, ***P < 0.001 vs. Control). (B) Oil red O staining of GEnCs treated with different concentrations of cholesterol (×400, bar=50μm). (C) Cholesterol quantification experiment was conducted to determine the content of intracellular cholesterol (*P < 0.05 vs. Control; **P < 0.01 vs. Control). (D) Expression of ABCA1 in GEnCs with different cholesterol concentrations (**P < 0.01 vs. Control, ***P < 0.001 vs. Control). (E,F) Expression of ABCA1 after intervention with different glucose concentrations without cholesterol and under cholesterol load (*P < 0.05 vs. Control). (G) Oil red O staining of GEnCs treated with different concentrations of cholesterol and glucose (×400, bar=50μm). (H) Cholesterol quantification experiment was conducted to determine the content of intracellular cholesterol (*P < 0.05 vs. LG, **P < 0.01 vs. LG; ***P < 0.001vs. LG). (I) RT-qPCR analysis to determine ABCA1 mRNA expression (***P < 0.001 vs. LG; #P < 0.05 vs. HC). (J) Western Blot analysis to determine ABCA1 protein expression (***P < 0.001 vs. LG; #P < 0.05 vs. HC). (K) Immunofluorescence experiment was conducted to determine the expression of ABCA1 (×200, bar=100μm).

**Figure 2. HG exacerbates intracellular cholesterol accumulation and pyroptosis under cholesterol load.** (A,B) RT-qPCR and Western Blot analysis to determine expressions of caspase-1, GSDMD, IL-1β in GEnCs under the condition of high glucose or/and high cholesterol (*P < 0.05 vs. LG,**P < 0.01 vs. LG; #P < 0.05 vs. HC, ##P < 0.01 vs. HC). (C) The release of LDH of GEnCs treated with different concentrations of cholesterol and glucose ((*P < 0.05 vs. LG, **P < 0.01 vs. LG; ##P < 0.01 vs. HC). (D,E) RT-qPCR and Western Blot analysis to determine ABCA1 expression of GEnCs treated with different concentrations of DIDS (*P < 0.05 vs. Control,***P < 0.001 vs. Control). (F) Oil red O staining of GEnCs treated with DIDS (×400, bar=50μm). (G) Cholesterol quantification experiment was conducted to determine intracellular cholesterol content (*P < 0.05 vs. Control). (H,I) RT-qPCR and Western Blot analysis to determine expressions of caspase-1, GSDMD, IL-1β in GEnCs treated with DIDS (*P < 0.05 vs. Control; **P < 0.01 vs. Control, ***P < 0.001 vs. Control). (J) Release of LDH of GEnCs treated with DIDS (****P < 0.001 vs. Control).

**Figure 3. Circ8411 is involved in the regulation of ABCA1.** (A) The heatmap for the differential expression of circRNAs. (B) RT-qPCR analysis to determine the expression of four candidate lncRNAs (*P < 0.05 vs. HC; **P < 0.01 vs. HC). (C) Circ8411 expression of GEnCs treated with cholesterol and glucose (***P < 0.001 vs. LG, ###P < 0.001 vs. HC). (D) RT-qPCR to determine the knockdown efficiency of circ8411 (*P < 0.05 vs. si-NC). (E,F) Oil red O staining of GEnCs (×400, bar=20μm) and cholesterol quantification experiment to determine the cholesterol accumulation in cells transfected with siRNA. (G-I) RT-qPCR, Western Blot, and immunofluorescence experiment (×200, bar=50μm) were conducted to determine the expression of ABCA1 in cells transfected with siRNA (*P < 0.05 vs. NC-si; **P < 0.01 vs. NC-si). (J.K) RT-qPCR and Western Blot analysis to determine expressions of caspase-1, GSDMD, IL-1β in GEnCs treated with siRNA (*P < 0.05 vs. NC-si; **P < 0.01 vs. NC-si, ***P < 0.001 vs. NC-si). (L) Release of LDH of GEnCs treated with siRNA (*P < 0.05 vs. NC-si). (M) RT-qPCR analysis to determine the overexpression efficiency of circ8411 (***P < 0.001 vs. vector); (N,O) Oil red O staining of GEnCs (×400, bar=20μm) and cholesterol quantification experiment to determine the cholesterol accumulation in cells transfected with circ8411-pcDNA3.1 plasmid (**P < 0.01 vs. vector). (P,Q,S) RT-qPCR, Western Blot, and immunofluorescence experiment (×200, bar=50μm) were conducted to determine the expression of ABCA1 in cells transfected with the circ8411-pcDNA3.1 plasmid (*P < 0.05 vs. vector, **P < 0.01 vs. vector). (R,S) RT-qPCR and Western Blot analysis to determine the expression of caspase-1, GSDMD, and IL-1β in GEnCs treated with circ8411-pcDNA3.1 plasmid (*P < 0.05 vs. LG; **P < 0.01 vs. LG). (T) Release of LDH of GEnCs treated with circ8411-pcDNA3.1 plasmid (*P < 0.05 vs. vector).

**Figure 4. The circ8411 regulates ABCA1 expression by regulating miR-23a-5p**

(A) RT-qPCR analysis to determine miR-23a-5p in the GEnCs under conditions of high glucose and high cholesterol. (B,C) RT-qPCR analysis to determine miR-23a-5p expression with the knockdown and overexpression of circ8411. (**P < 0.05 vs. Control). (D,E) RT-qPCR analysis to determine the overexpression efficiency of miR-23a-5p and the expression of ABCA1 in cells transfected with the miR-23a-5p mimic (**P < 0.05 vs. Control). (F,G) Rescue experiments to determine the regulatory effect of circ8411/miR-23a-5p/ABCA1 in GEnCs (*P < 0.05 vs. mimic NC; #P < 0.05 vs. miR-23a-5p mimic with circ8411 vector). (H) Luciferase reporter assay on GEnCs co-transfected with circ8411-3’UTR-WT or circ8411-3’UTR-MUT and miR-23a-5p mimics or NC (**P < 0.01 vs. Circ8411-WT+NC group).

**Figure 5. High glucose affects circ8411 down-regulation through RXRs.** (A) RT-qPCR analysis to determine the expression of RXRα, CEBP/α, Hif-1a in the GEnCs undering glucose and cholesterol (*P < 0.05 vs. Control, **P < 0.01 vs. Control). (B) Western Blot showed the expression of RXRα GEnCs under different intervention of glucose and cholesterol (*P < 0.05 vs. Control). (C) RXRα expression of GEnCs treated with different concentrations of UVI3003 (*P < 0.05 vs. Control). A marked reduction in RXRα was observed in cells treated with 10umol/L of UVI3003. (D) Circ8411 expression of GEnCs treated with UVI3003. (E) ABCA1 expression of GEnCs treated with UVI3003 (*P < 0.05 vs. Control).

**Figure 6. GLP-1RA attenuated cholesterol accumulation and pyroptosis in GEnCs by increasing RXRα-circ8411-miR-23a-5p-ABCA1.** (A,B) CCK-8 assay was used to evaluate the influence of liraglutide and loxenatide on GEnC viability (***P < 0.001 vs. Control). (C,D) ABCA1 expression in the GEnCs treated with different concentrations of liraglutide and loxenatide (*P < 0.05 vs. Control; **P < 0.01 vs. Control). (E-H) Oil red O staining of GEnCs (×400, bar=50μm) and cholesterol quantification experiment to determine the cholesterol accumulation in cells treated with liraglutide and loxenatide (*P < 0.05 vs. Control). (I,J,L,M) Expressions of caspase-1, GSDMD, IL-1β in GEnCs treated with liraglutide and loxenatide (*P < 0.05 vs. Control; **P < 0.01 vs. Control). (K,N) Release of LDH of GEnCs treated with liraglutide and loxenatide (**P < 0.01 vs. Control). (O-T) Expressions of RXRα, circ8411, and miR-23a-5p of GEnCs treated with liraglutide and loxenatide (*P < 0.05 vs. Control, **P < 0.01 vs. Control). (U-V) Western Blot was performed to determine the ABCA1 expression in GEnCs treated with siRNAof circ8411 and GLP-1RA (*P < 0.05 vs. Control).

**Figure 7. GLP-1RA alleviated kidney damage, cholesterol accumulation, and pyroptosis in the kidneys of *ApoE^-/-^*DM mice by increasing circ8411-ABCA1.** (A) HE staining of the kidney tissues of mice (×400, bar=50μm). (B) Oil red O staining of the kidney tissues of mice (×400, bar=50μm). (C) Cholesterol quantification experiment to determine the cholesterol accumulation in the kidneys of mice (**P < 0.01 vs. WT-NC, ***P < 0.001 vs. WT-NC, #P < 0.05 vs. *ApoE^-/-^*DM, ##P < 0.01 vs. *ApoE^-/-^*DM).(D,E) Caspase-1, GSDMD, IL-1β expression in the kidneys of mice (*P < 0.05 vs. WT-NC, **P < 0.01 vs. WT-NC, ***P < 0.001 vs. WT-NC, #P < 0.05 vs. *ApoE^-/-^*DM, ##P < 0.01 vs. *ApoE^-/-^*DM). (F) Release of LDH in the kidneys of mice (**P < 0.01 vs. WT-NC, ##P < 0.01 vs. *ApoE^-/-^*DM). (G) Circ8411 expression of renal of mice (*P < 0.05 vs. WT-NC, ##P < 0.01 vs. *ApoE^-/-^*). (H-J) RT-qPCR, Western Blot, and immunofluorescence experiment (×200, bar=50μm) were conducted to determine ABCA1(red) expression in the kidneys of mice. Nuclei were stained with DAPI (blue). (**P < 0.01 vs. WT-NC, ***P < 0.001 vs. WT-NC, ##P < 0.01 vs. *ApoE^-/-^*, ###P < 0.001 vs. *ApoE^-/-^*, &P < 0.05 vs. WT-NC).

Once again, thank you very much for your constructive comments and suggestions which would help us both in English and in depth to improve the quality of the paper.

Yours sincerely,

Pei Yu

2024/10/16
