# Supplementary material for: GLP-1RA improves diabetic renal injury by alleviating glomerular endothelial cells pyrotosis via RXRα/circ8411/miR-23a-5p/ABCA1 pathway
Source: PLoS One. 2024 Dec 2;19(12):e0314628. doi: 10.1371/journal.pone.0314628 (PMC11611192; doi:10.1371/journal.pone.0314628)
Supplement: Attachment — Submitted filename: Response to editor and reviewers.docx [file pone.0314628.s006.docx]

Dear editor and reviewers,

We would like to express our sincere gratitude to you for your efforts in handling our manuscript. We appreciate you give us the opportunity to revise our paper. We thank the editorial boards and the reviewer’s points and suggestions to improve our work. Those comments are all valuable and very helpful for revising and improving our paper.

We have studied comments carefully and have made correction which we hope meet with approval. Based on your comment and request, we attached revised manuscript with the correction sections red marked. The corrections in the paper and the responds to the reviewer’s comments are as following.

Should you have any questions, please contact us without hesitate.

**“While I appreciate the improvements made I have some concerns regarding its readiness for publication. The language still presents challenges, with several grammatical errors that affect clarity. Some sentences in the manuscript (in particular, in the introduction) are quite short and lead to difficulty in following the overall narrative. I recommend rephrasing them for better flow and coherence. In addition there are also ongoing inconsistencies in terminology and figure captions, which complicate the evaluation of the findings.**

**I recommend a thorough revision to address these issues before considering acceptance, as the article has interesting potential. ”**

**Response:** We apologize for the presence of grammatical and typographical errors in our article, as they should have been avoided. In order to refine the language and rectify any remaining errors, we have submitted them to a professional editing service. We attached revised manuscript with the correction sections red marked. Based on your suggestions, we have thoroughly examine and revise the figure captions and the section of introduction.Additionally, we have re-edited the figures with the objective of enhancing clarity and comprehensibility.

**Revised version in line 26,29,30,36,38,42,49(Abstract) :**

Lipotoxicity has been implicated in diabetic kidney disease (DKD). However, the role of high glucose levels in DKD and the underlying renal protective mechanisms of GLP-1 receptor agonists (GLP-1RAs) remain unclear.

To investigate cholesterol accumulation, pyroptosis in glomerular endothelial cells (GEnCs), and the renal protective mechanisms of GLP-1RAs, we used various techniques, including RT-qPCR, Oil Red O staining, Western blotting, lactate dehydrogenase (LDH) activity assays, circRNA microarrays, bioinformatics analysis, gain and loss-of-function experiments, rescue experiments, and luciferase assays.

GEnCs exposed to high glucose exhibited reduced cholesterol efflux, which was accompanied by downregulation of ATP-binding cassette transporter A1 (ABCA1) expression, cholesterol accumulation, and pyroptosis. Circ8411 was identified as a regulator of ABCA1, inhibiting miR-23a-5p through its binding to the 3'UTR. Additionally, higher glucose levels decreased circ8411 expression by inhibiting RXRα. GLP-1RAs effectively reduced cholesterol accumulation and cell pyroptosis by targeting the RXRα/circ8411/miR-23a-5p/ABCA1 pathway. In diabetic *ApoE^-/-^* mice, renal structure and function were impaired, with resulted in increased cholesterol accumulation and pyroptosis; however, GLP-1RAs treatment reversed these detrimental changes.

Moreover, GLP-1RAs may provide reno-protective effects by activating this pathway.

**Revised version in line (Introduction) :**

Diabetic kidney disease (DKD) is a leading cause of end-stage renal disease (ESRD) [1],which poses a significant health and economic burden [2]. Current strategies to prevent or treat DKD have limited efficacy, such as the management of blood glucose, blood pressure, and urinary protein excretion. This limitation can be attributed to an insufficient understanding of the underlying pathophysiological mechanisms [3].

Numerous studies have established a strong association between abnormal lipid metabolism and DKD. The accumulation of triglycerides(TG) and cholesterol in the kidneys has been implicated in the pathogenesis of DKD [4-6]. Lipotoxicity, which is characterized by the dysregulation of intracellular homeostasis due to lipid accumulation, leads to metabolic, inflammatory, and oxidative stress [7]. Hyperlipidemia, oxidized low-density lipoprotein (ox-LDL), and cholesterol crystals have been shown to activate caspase-1 in endothelial cells, thereby triggering pyroptosis [8, 9]. Pyroptosis is an inflammatory process that is characterised by the release of pro-inflammatory factors, including interleukin-1β (IL-1β), IL-18, and lactate dehydrogenase (LDH). These factors can contribute to a more pronounced inflammatory response[10]. However, the specific mechanisms underlying pyroptosis in the context of DKD development remain unclear.

There is an increasing consensus that glomerular endothelial cells (GEnCs) play a critical role in the development and progression of DKD [11-13]. The ATP-binding cassette transporter A1 (ABCA1) is a key regulator of cholesterol efflux from cells, promoting the removal of cholesterol [14-16]. Previous researches suggest that ABCA1 expression in GEnCs may help to prevent endothelial dysfunction [5, 17].

ABCA1 is a plasma membrane protein that serves as a key regulator of cholesterol efflux from cells[18, 19]. *In vitro* studies have demonstrated that stimulation of high glucose can downregulate ABCA1 expression in renal cells, and a similar decrease in ABCA1 has been observed in diabetic and nephrotic mouse models [20, 21]. Furthermore, our previous research indicated that the dysregulation of glucose and cholesterol metabolism can impair ABCA1 function [22]. Collectively, these findings suggest that dysregulation of ABCA1 may contribute to cholesterol accumulation in the kidney.

Noncoding RNAs (ncRNAs) are a class of RNAs that do not encode proteins. A specific subset of ncRNAs are known as circular RNAs (circRNAs). CircRNAs are characterized by a covalently closed loop formed through a back-splicing process [23]. CircRNAs can act as microRNA (miRNA) sponges or interact with RNA-binding proteins, thereby regulating gene expression [24]. They have been identified as key regulators in various diseases [25]. However, the potential role of circRNAs in the progression of DKD remains unexplored. The glucose-like peptide-1 receptor (GLP-1R) and its agonists (GLP-1RAs) have shown protective effects against oxidative stress and macrophage activation in DKD [26]. Specifically, Exendin-4, a GLP-1RA, has been shown to ameliorate lipotoxicity-induced injury in GEnCs by enhancing ABCA1-mediated cholesterol efflux [27, 28]. Based on these findings, we hypothesize that GLP-1RAs may exert their renal protective effects in DKD by regulating lipotoxicity-induced pyroptosis in GEnCs through the modulation of ABCA1. Notably, circRNAs may play a significant role in this regulatory process.

**Revised version in Results(238-254) :**

CCK8 assay and Oil Red O staining were employed to determine the optimal concentration and time for cholesterol. Based on the results, 400 μg/ml cholesterol and 24 hours were identified as optimal intervention concentration and time for GEnCs(Fig 1A-B). The treatment of GEnCs with varying concentrations of glucose and cholesterol demonstrated a gradual elevation in ABCA1 mRNA expression with increasing cholesterol levels(Fig 1D), and the reduction in ABCA1 mRNA was most pronounced when cells were treated with 25.5mmo/L glucose(Fig 1E-F). Accordingly, 400 µM cholesterol and 25 µM glucose were selected as the intervention concentrations for the subsequent cell experiments. When compared to high glucose or high cholesterol individually, intracellular cholesterol accumulation was significantly elevated in cells treated with a combination of high glucose and high cholesterol (Fig 1G, H). Furthermore, the results demonstrated that ABCA1 levels were downregulated in the HG group compared to LG and significantly upregulated in the HC group. ABCA1 expression was significantly downregulated in the HG and HC groups compared to HC (Fig 1I-K). In summary, these findings suggest that high glucose may exacerbate intracellular cholesterol accumulation under conditions of cholesterol overload, the process may be mediated by ABCA1.

**Revised version in Results(419-421) :**

Among these, CEBP/α and RXRα exhibited significantly decreased expression in GEnCs cultured in HG+HC group compared to those cultured in HC group (Fig 5A). RXRα was selected for further investigation in the subsequent study. To suppress RXRα expression effectively in GEnCs, we employed different concentrations of UVI3003.

**Revised version in Captions：**

**Figure 1. High glucose exacerbates intracellular cholesterol accumulation under cholesterol load in GEnCs.** (A) CCK-8 assay was used to evaluate the influence of water-soluble cholesterol on GEnCs viability (*P < 0.05 vs. Cho-0μg/ml group; **P < 0.01 vs. Cho-0μg/ml group, ***P < 0.001 vs. Cho-0μg/ml group). (B)Representative images of Oil red O staining of GEnCs treated with different concentrations of cholesterol and the quantitative assessment(×400, bar=50μm; ***P < 0.001 vs. Cho-0μg/ml group). (C) Cholesterol quantification experiment was conducted to determine the content of intracellular cholesterol (*P < 0.05 vs. Cho-0μg/ml group; **P < 0.01 vs. Cho-0μg/ml group). (D)The mRNA expression of ABCA1 in GEnCs was detected by RT-qPCR with different cholesterol concentrations (**P < 0.01 vs. Cho-0 μg/ml group, ***P < 0.001 vs. Cho-0μg/ml group). (E) The mRNA expression of ABCA1 in GEnCs was detected by RT-qPCR after intervention with different glucose concentrations without cholesterol(*P < 0.05 vs. Glu-5.5mmol/L group). (F) The mRNA expression of ABCA1 in GEnCs was detected by RT-qPCR after intervention with different glucose concentrations under cholesterol load (#P < 0.05 vs. Cho-400μg/ml+Glu-5.5mmol/L group). (G) Representative images of Oil red O staining of GEnCs and the quantitative assessment(×400, bar=50μm; ##P < 0.01 vs. HC, ***P < 0.001 vs. LG group). (H) Cholesterol quantification experiment was conducted to determine the content of intracellular cholesterol (*P < 0.05 vs. LG group, **P < 0.01 vs. LG group; ***P < 0.001vs. LG group). (I) RT-qPCR analysis to determine ABCA1 mRNA expression (***P < 0.001 vs. LG group; #P < 0.05 vs. HC group). (J) Western Blot analysis to determine ABCA1 protein expression (***P < 0.001 vs. LG group; #P < 0.05 vs. HC group). (K) Representative images of Immunofluorescence staining of ABCA1(red) in GEnCs(×200, bar=100μm). Nucleus were stained with DAPI (blue).

**Figure 2. HG exacerbates GEnCs pyroptosis through ABCA1 under cholesterol load.** (A,B) RT-qPCR and Western Blot analysis to determine expressions of caspase-1, GSDMD, IL-1β in GEnCs under the condition of high glucose or/and high cholesterol (*P < 0.05 vs. LG group,**P < 0.01 vs. LG group; #P < 0.05 vs. HC group, ##P < 0.01 vs. HC group). (C) The release of LDH of GEnCs treated with different conditions(*P < 0.05 vs. LG group, **P < 0.01 vs. LG group; ##P < 0.01 vs. HC group). (D,E) RT-qPCR and Western Blot analysis to determine ABCA1 expression of GEnCs treated with different concentrations of DIDS (*P < 0.05 vs. HG+HC+DIDS-0μmmol/L group,***P < 0.001 vs. HG+HC+DIDS-0μmmol/L group). (F) Representative images of Oil red O staining and analysis of GEnCs treated with 400 μmol/L DIDS (×400, bar=50μm；**P < 0.01 vs. HG+HC group). (G) Cholesterol quantification experiment was conducted to determine intracellular cholesterol content (*P < 0.05 vs. HG+HC group). (H,I) RT-qPCR and Western Blot analysis to determine expressions of caspase-1, GSDMD, IL-1β in GEnCs treated with 400 μmol/L DIDS (*P < 0.05 vs. HG+HC group; **P < 0.01 vs. HG+HC group, ***P < 0.001 vs. HG+HC group). (J) Release of LDH of GEnCs treated with 400 μmol/L DIDS (***P < 0.001 vs. HG+HC group).

**Figure 3. Circ8411 is involved in the regulation of ABCA1.** **Figure 3. Circ8411 is involved in the regulation of ABCA1.** (A) The heatmap for the differential expression of circRNAs. (B) RT-qPCR analysis to determine the expression of four candidate lncRNAs (*P < 0.05 vs. HC group; **P < 0.01 vs. HC group). (C) Circ8411 expression of GEnCs treated with cholesterol and glucose (***P < 0.001 vs. LG group, ###P < 0.001 vs. HC group). (D) RT-qPCR to determine the knockdown efficiency of circ8411 (*P < 0.05 vs. NC-si group). (E) Representative images of Oil red O staining(×400, bar=20μm) and analysis in GEnCs after knockedown circ8411(***P < 0.001 vs. HG+HC+si-NC group). (F) Cholesterol quantification experiment to determine the cholesterol accumulation in GEnCs transfected with siRNA (*P < 0.05 vs. HG+HC+si-NC group). (G-I) RT-qPCR, Western Blot, and immunofluorescence experiment (×200, bar=50μm) were conducted to determine the expression of ABCA1 in cells transfected with siRNA (*P < 0.05 vs. NC-si group; **P < 0.01 vs. NC-si group). (J) RT-qPCR analysis to determine expressions of caspase-1, GSDMD, IL-1β in GEnCs treated with siRNA (*P < 0.05 vs. NC-si group; **P < 0.01 vs. NC-si group, ***P < 0.001 vs. NC-si group). (K)Representative Western blot images and quantitative data of caspase-1, GSDMD, IL-1β in GEnCs treated with siRNA (*P < 0.05 vs. HG+HC+si-NC group; **P < 0.01 vs. HG+HC+si-NC group, ***P < 0.001 vs. HG+HC+si-NC group). (L) Release of LDH in GEnCs treated with siRNA (*P < 0.05 vs. HG+HC+si-NC group). (M) RT-qPCR analysis to determine the overexpression efficiency of circ8411 (***P < 0.001 vs. cir8411-vector group). (N) Representative images of Oil red O staining(×400, bar=20μm) and analysis in GEnCs transfected with circ8411-pcDNA3.1 plasmid(**P < 0.01 vs. HG+HC+cir8411-vector group). (O) Cholesterol quantification experiment to determine the cholesterol accumulation in GEnCs transfected with circ8411-pcDNA3.1 plasmid (**P < 0.01 vs. HG+HC+cir8411-vector group). (P) RT-qPCR were conducted to determine the expression of ABCA1 mRNA in cells transfected with the circ8411-pcDNA3.1 plasmid (**P < 0.01 vs. HG+HC+cir8411-vector group). (Q)Representative images of the immunofluorescence staining of ABCA1(red) in cells transfected with the circ8411-pcDNA3.1 plasmid (×200, bar=50μm). Nucleus were stained with DAPI (blue). (R) RT-qPCR analysis to determine the expression of caspase-1, GSDMD, and IL-1β in GEnCs treated with circ8411-pcDNA3.1 plasmid (*P < 0.05 vs. cir8411-vector group, **P < 0.01 vs. cir8411-vector group). (S) Representative Western blot images and quantitative data of caspase-1, ABCA1, GSDMD, and IL-1β in GEnCs treated with circ8411-pcDNA3.1 plasmid (*P < 0.05 vs. HG+HC+cir8411-vector group; **P < 0.01 vs. HG+HC+cir8411-vector group). (T) Release of LDH in GEnCs treated with circ8411-pcDNA3.1 plasmid (*P < 0.05 vs. HG+HC+cir8411-vector group).

**Figure 4. The circ8411 regulates ABCA1 expression by regulating miR-23a-5p**

(A) RT-qPCR analysis to determine miR-23a-5p in the GEnCs under different conditions (**P < 0.01 vs. LG group；###P < 0.001 vs. HG group). (B) RT-qPCR analysis to determine miR-23a-5p expression with the knockdown of circ8411(**P < 0.01 vs. LG+si-NC group, ##P < 0.01 vs. HG+HC+si-NC group). (C) RT-qPCR analysis to determine miR-23a-5p expression with the overexpression of circ8411 (**P < 0.01 vs. LG+circ8411-vector group, ##P < 0.01 vs. HG+HC+circ8411-vector group). (D) RT-qPCR analysis to determine the overexpression efficiency of miR-23a-5p in cells treated with the miR-23a-5p mimic (***P < 0.001 vs. miR-23a-5p mimic-NC group). (E) RT-qPCR analysis to determine the expression of ABCA1 in cells treated with the miR-23a-5p mimic (**P < 0.01 vs. HG+HC+mimic-NC group). (F) Rescue experiments to determine the regulatory effect of circ8411/miR-23a-5p/ABCA1 in GEnCs (*P < 0.05 vs. HG+HC+mimic-NC group, #P < 0.05 vs. HG+HC+mimic-miR-23a-5p+circ8411 vector group). (G) Western blot analysis to determine expression of ABCA1 in rescue experiments(*P < 0.05 vs. HG+HC+mimic-NC group; #P < 0.05 vs. HG+HC+mimic-miR-23a-5p+circ8411 vector group). (H) Luciferase reporter assay on GEnCs co-transfected with circ8411-3’UTR-WT or circ8411-3’UTR-MUT and miR-23a-5p mimic or NC (**P < 0.01 vs. mimic-NC group).

**Figure 5. High glucose affects circ8411 down-regulation through RXR**α**.**

(A) RT-qPCR analysis to determine the expression of RXRα, CEBP/α, Hif-1a in the GEnCs undering glucose and cholesterol (*P < 0.05 vs. HC group,**P < 0.01 vs. HC group). (B) The mRNA expression of RXRα in GEnCs treated with different concentrations of UVI3003 (*P < 0.05 vs. UVI3003-0μmol/L group). (C) The mRNA expression of Circ8411 in GEnCs treated with 10 μmol/L UVI3003(**P < 0.01 vs. NC group). (D)The mRNA expression of ABCA1 expression in GEnCs treated with 10 μmol/L UVI3003 (*P < 0.05 vs. NC group). (E) Representative Western blot images and quantitative data of ABCA1 in GEnCs treated with 10 μmol/L UVI3003(**P < 0.01 vs. UVI3003-0μmol/L group).

**Figure 6. GLP-1RA attenuated cholesterol accumulation and pyroptosis in GEnCs by increasing RXRα-circ8411-miR-23a-5p-ABCA1.** (A,B) CCK-8 assay was used to evaluate the influence of liraglutide and loxenatide on GEnC viability (***P < 0.001 vs. HG+HC+Loxe-0nmol/L group, ###P < 0.001 vs. HG+HC+Lira-0nmol/L group). (C) ABCA1 expression in the GEnCs treated with different concentrations of liraglutide (#P < 0.05 vs. HG+HC+Lira-0nmol/L group, ##P < 0.01 vs. HG+HC+Lira-0nmol/L group, ###P < 0.001 vs. HG+HC+Lira-0nmol/L group). (D) ABCA1 expression in the GEnCs treated with different concentrations of loxenatide (***P < 0.001 vs. HG+HC+Loxe-0nmol/L group). (E,G) Representative images of Oil red O staining(×400, bar=20μm) and quantitative analysis in GEnCs treated with liraglutide and loxenatide(*P < 0.05 vs. HG+HC group, **P < 0.01 vs. HG+HC group). (F,H) Cholesterol quantification experiment to determine the cholesterol accumulation in cells treated with liraglutide and loxenatide (*P < 0.05 vs. HG+HC group). (I) The mRNA expression of caspase-1, GSDMD, IL-1β in GEnCs treated with liraglutide (**P < 0.01 vs. HG+HC group). (J) Representative Western blot images and quantitative data of caspase-1, GSDMD, N-GSDMD, IL-1β in GEnCs treated with loxenatide (*P < 0.05 vs. HG+HC group, **P < 0.01 vs. HG+HC group). (L) The mRNA expression of caspase-1, GSDMD, IL-1β in GEnCs treated with loxenatide (*P < 0.05 vs. HG+HC group, **P < 0.01 vs. HG+HC group). (M) Representative Western blot images and quantitative data of caspase-1, GSDMD, N-GSDMD, IL-1β in GEnCs treated with liraglutide (*P < 0.05 vs. HG+HC group, **P < 0.01 vs. HG+HC group). (K,N) Release of LDH of GEnCs treated with loxenatide and liraglutide (*P < 0.05 vs. HG+HC group, **P < 0.01 vs. HG+HC group). (O-T) Expressions of RXRα, circ8411, and miR-23a-5p of GEnCs treated with liraglutide and loxenatide (*P < 0.05 vs. HG+HC group, **P < 0.01 vs. HG+HC group). (U) Representative images of Western blot and quantitative data of the ABCA1 expression in GEnCs treated with si-RNA of circ8411 and liraglutide (*P < 0.05 vs. HG+HC+Lira+si-NC group).(V) Representative images of Western blot and quantitative data of the ABCA1 expression in GEnCs treated with si-RNA of circ8411 and loxenatide (*P < 0.05 vs. HG+HC+Loxe+si-NC group).

**Figure 7. GLP-1RA alleviated kidney damage, cholesterol accumulation, and pyroptosis in the kidneys of *ApoE^-/-^*DM mice by increasing circ8411-ABCA1.** (A) Representative images of HE staining of the kidney tissues of mice (×400, bar=50μm). (B) Representative images of Oil red O staining and the quantitative assessment of the kidney tissues of mice (×400, bar=50μm, **P < 0.01 vs. WT-NC group, ***P < 0.001 vs. WT-NC group, ###P < 0.001 vs. *ApoE^-/-^*DM group). (C) Cholesterol quantification experiment to determine the cholesterol accumulation in the kidneys of mice (**P < 0.01 vs. WT-NC group, ***P < 0.001 vs. WT-NC group, ##P < 0.01 vs. *ApoE^-/-^*DM group).(D) The mRNA expression of caspase-1, GSDMD, IL-1β in the kidneys of mice (*P < 0.05 vs. WT-NC group, **P < 0.01 vs. WT-NC group,***P < 0.001 vs. WT-NC group, #P < 0.05 vs. *ApoE^-/-^*DM group, ##P < 0.01 vs. *ApoE^-/-^*DM group, ###P < 0.001 vs. *ApoE^-/-^*DM group). (E) Representative Western blot images and quantitative data of of caspase-1, GSDMD, IL-1β in the kidneys of mice (*P < 0.05 vs. WT-NC group, **P < 0.01 vs. WT-NC group, #P < 0.05 vs. *ApoE^-/-^*DM group).(F) Release of LDH in the kidneys of mice (**P < 0.01 vs. WT-NC group, ##P < 0.01 vs. *ApoE^-/-^*DM group). (G) Circ8411 expression of renal of mice (*P < 0.05 vs. WT-NC group, ##P < 0.01 vs. *ApoE^-/-^* group, &P < 0.05 vs. *ApoE^-/-^*DM group). (H) miR-23a-5p expression of renal of mice (**P < 0.01 vs. WT-NC group,***P < 0.001 vs. WT-NC group, &P < 0.05 vs. *ApoE^-/-^*DM group). (I-J) RT-qPCR and Western Blot experiment were conducted to determine ABCA1 expression in the kidneys of mice (**P < 0.01 vs. WT-NC group, ***P < 0.001 vs. WT-NC group, ##P < 0.01 vs. *ApoE^-/-^* group, &P < 0.05 vs. *ApoE^-/-^*DM group). (K) Representative images of the immunofluorescence staining(×200, bar=50μm) of ABCA1(red) in the kidneys of mice. Nucleus were stained with DAPI (blue).

**Figure 8. Proposed model for the signaling pathway by which high glucose participates in the GEnCs injury via the RXRα/circ8411/miR-23a-5p/ABCA1 pathway.** High glucose decreased the ability of GEnCs to respond to cholesterol load, leading to downregulation of ABCA1 expression, increased cholesterol accumulation, and pyroptosis in GEnCs. Circ8411 regulated ABCA1 by inhibiting the action of miR-23a-5p, and miR-23a-5p binds to the 3' UTR of circ8411. High glucose could decrease circ8411 expression by inhibiting RXRα. GLP-1RAs could potentially reduce cholesterol accumulation and cell pyroptosis by modulating the RXRα/circ8411/miR-23a-5p/ABCA1 axis.

Once again, thank you very much for your constructive comments and suggestions which would help us both in English and in depth to improve the quality of the paper.

Yours sincerely,

Pei Yu

2024/11/11
